# Sequential Bilateral Optic Neuropathy Associated With IgM-Secreting B-cell Non-Hodgkin Lymphoma

**DOI:** 10.7759/cureus.117814

**Published:** 2026-10-12

**Authors:** Min Ju Kang, Axel L Rivas, Jasmin Kumar, Paul Janda, Aroucha Vickers

**Affiliations:** 1 Medicine, Kirk Kerkorian School of Medicine at UNLV, Las Vegas, USA; 2 Ophthalmology, The University of Texas at Austin, Austin, USA; 3 Neurology, Valley Hospital Medical Center, Las Vegas, USA; 4 Neurology, Las Vegas Neurology Center, Las Vegas, USA

**Keywords:** bilateral optic neuropathy, lymphoma-associated optic neuropathy, monoclonal gammopathy, non-hodgkin lymphoma, optic neuropathy, waldenström macroglobulinemia, zanubrutinib

## Abstract

Optic neuropathy associated with hematologic malignancy may present with heterogeneous clinical and radiographic findings, creating diagnostic uncertainty. We report the case of a 76-year-old man with vascular risk factors who developed new-onset, painless vision loss in the right eye accompanied by optic disc edema. Cerebral angiography identified a dural arteriovenous (AV) fistula with feeding-artery aneurysms, and subsequent evaluation demonstrated left internal carotid artery stenosis, presenting competing vascular explanations during the diagnostic evaluation. Vision did not improve following vascular intervention or high-dose corticosteroid therapy, and two orbital MRI examinations revealed no abnormalities.

Approximately one year later, the patient developed new vision loss in the previously unaffected left eye, with sequential optic disc edema followed by bilateral optic atrophy and severe visual-field loss. Expanded systemic evaluation revealed an IgM monoclonal gammopathy, and bone marrow biopsy demonstrated CD5-/CD10- B-cell non-Hodgkin lymphoma involving 20-25% of marrow cellularity. Following the initiation of zanubrutinib, serum IgM levels declined; however, objective visual fields remained severely depressed despite subjective improvement in daytime vision. Although an association between the lymphoproliferative disorder and optic neuropathy is biologically plausible, the available clinical data do not establish causation or a definitive mechanism of optic nerve injury, and competing etiologies could not be completely excluded. This report highlights the importance of continued systemic evaluation when sequential optic neuropathy remains unexplained despite nondiagnostic neuroimaging. In selected patients, hematologic evaluation, including serum protein electrophoresis, may identify an underlying monoclonal gammopathy or lymphoproliferative disorder.

## Introduction

Optic neuropathy, or dysfunction of the optic nerve, is a major cause of visual loss and has a broad differential diagnosis, including ischemic, inflammatory, compressive or infiltrative, toxic or nutritional, and hereditary causes [1]. In older adults with vascular risk factors, non-arteritic anterior ischemic optic neuropathy (NAION) is an important consideration when evaluating acute, painless optic neuropathy. Progressive optic neuropathy, particularly when the clinical course is atypical, or the initial diagnostic evaluation is unrevealing, should prompt consideration of less common systemic or infiltrative processes [1-2].

Lymphoma-associated optic neuropathy is an uncommon neuro-ophthalmic manifestation of hematologic malignancy. Optic neuropathy has also been described in association with IgM-secreting lymphoproliferative disorders, including through proposed paraproteinemic, immune-mediated, and hyperviscosity-related mechanisms that do not require direct optic nerve infiltration [3-4]. The clinical presentation is heterogeneous, and diagnosis can be challenging because neuroimaging and other conventional diagnostic studies may be unrevealing, particularly early in the disease course [2, 5-6]. Delayed recognition may postpone treatment and contribute to irreversible visual loss.

We report a case of progressive sequential bilateral optic neuropathy that preceded the diagnosis of an IgM-secreting CD5-/CD10- B-cell non-Hodgkin lymphoma. The patient's evaluation was complicated by repeatedly unrevealing orbital MRI findings and competing vascular abnormalities, illustrating the diagnostic uncertainty that may arise when optic neuropathy and a systemic lymphoproliferative disorder occur concurrently.

## Case presentation

A 76-year-old man with a history of hypertension, prostate cancer in remission, left carotid endarterectomy, and former tobacco use presented with new-onset, painless vision loss in the right eye, described as peripheral visual difficulty and shadowing. He did not seek evaluation until several weeks after symptom onset, limiting precise characterization of the tempo of visual decline. Initial evaluation at an outside hospital revealed right optic disc edema. Cerebral angiography identified a left transverse sinus dural arteriovenous (AV) fistula with feeding-artery aneurysms arising from the left superior cerebellar artery. The patient underwent embolization of the fistula and coil embolization of the aneurysm, neither of which improved his vision. The overall clinical course, diagnostic evaluation, and treatment are summarized in Table 1.

**Table 1:** Clinical course, diagnostic evaluation, and management CTA: computed tomography angiography; GCA: giant cell arteritis; HVF: Humphrey visual field; ICA: internal carotid artery; MD: mean deviation; OCT: optical coherence tomography; RAPD: relative afferent pupillary defect; RNFL: retinal nerve fiber layer; SPEP: serum protein electrophoresis; VA: visual acuity

Relative time	Clinical course/ophthalmic findings	Evaluation/management
Initial presentation	New-onset painless right-eye peripheral visual difficulty and shadowing; evaluation occurred several weeks after symptom onset; right optic disc edema	Cerebral angiography identified left transverse sinus dural AV fistula with feeding artery aneurysms; embolization and coil embolization performed without visual improvement
~1 week after initial evaluation	Persistent right optic disc edema with disc hemorrhage; OCT RNFL 223 µm OD and 103 µm OS	General ophthalmology evaluation
~1 month after initial evaluation	VA 20/50 OD and 20/20 OS; right RAPD and grade 2 disc edema; HVF MD −23.23 dB OD and −3.50 dB OS; RNFL 115 µm OD and 106 µm OS	Neuro-ophthalmology evaluation; infectious/autoimmune workup unrevealing; GCA considered; prednisone 80 mg daily trial without visual improvement
~1 year after initial presentation	New left-eye vision loss; right optic disc pallor and new left disc edema; VA 20/40 OD and 20/50 OS; HVF MD −25.81 dB OD and −28.44 dB OS; RNFL 35 µm OD and 126 µm OS	CTA showed ~70% left ICA stenosis; MRI brain/orbits showed no optic nerve lesion or compression; expanded systemic evaluation initiated
Subsequent systemic evaluation	Progressive bilateral optic atrophy and severe visual-field loss	First documented SPEP demonstrated M-spike 0.6 g/dL; subsequent hematologic evaluation demonstrated IgM monoclonal gammopathy and CD5−/CD10− B-cell non-Hodgkin lymphoma
Following hematologic diagnosis	Severe bilateral optic neuropathy/atrophy	Zanubrutinib initiated; serial IgM levels declined
Post-treatment follow-up	Patient reported subjective improvement in daytime vision; VA 20/40−1 OD and 20/30 OS; HVF remained severely depressed (MD −29.51 dB OD, −30.14 dB OS)	Continued neuro-ophthalmic and hematologic follow-up

Approximately one week later, evaluation by a general ophthalmologist demonstrated right optic disc edema with an associated disc hemorrhage. Optical coherence tomography (OCT) demonstrated retinal nerve fiber layer (RNFL) thicknesses of 223 µm in the right eye and 103 µm in the left eye (normal average 95-115 µm) (Figure 1). Given the persistent optic disc edema, the patient was treated with high-dose oral prednisone (80 mg daily) without visual improvement and was subsequently referred for neuro-ophthalmologic evaluation.

**Figure 1:**
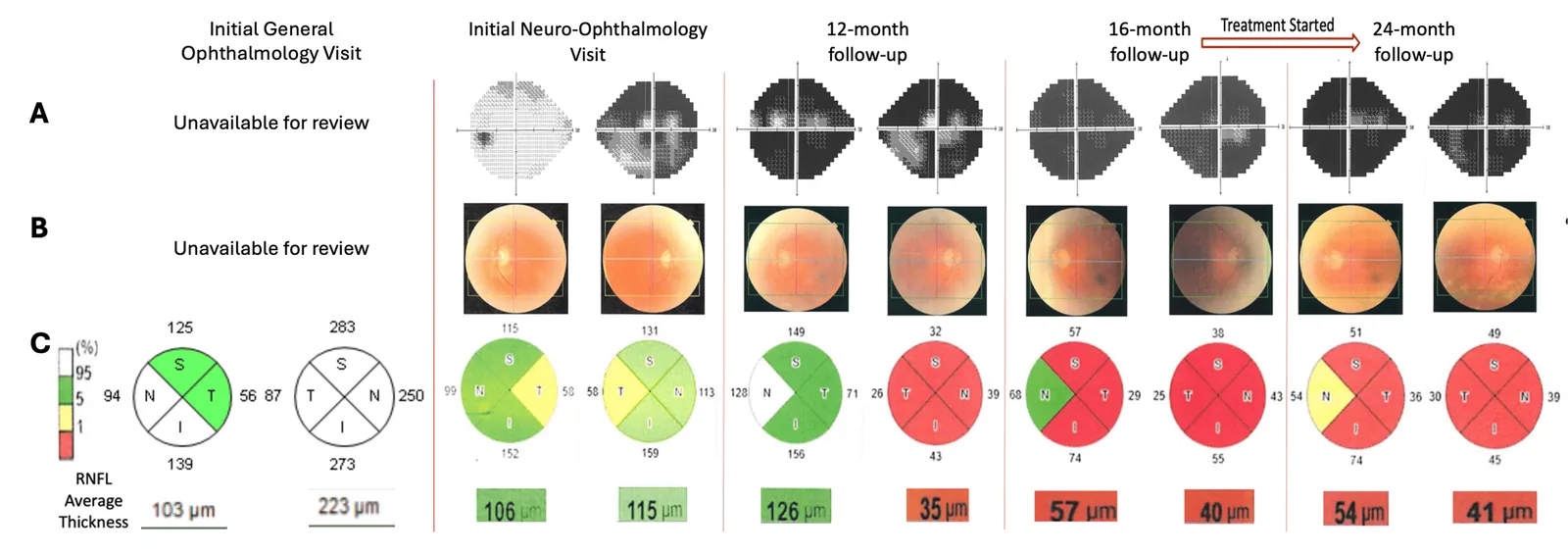
Serial visual field, fundus, and OCT findings during disease progression and treatment (A) Representative Humphrey visual fields demonstrating progressive visual field loss over serial follow-up. (B) Color fundus photographs demonstrating the evolution of optic disc changes during sequential bilateral involvement. (C) OCT RNFL analyses demonstrating initial RNFL thickening associated with optic disc edema followed by RNFL thinning corresponding to optic atrophy during sequential bilateral involvement. At the initial general ophthalmology visit, OCT demonstrated average RNFL thicknesses of 223 µm in the right eye and 103 µm in the left eye; visual field and fundus photographs from this visit were unavailable for review. Subsequent findings are shown at the initial neuro-ophthalmology visit and at the approximately 12-, 16-, and 24-month follow-up. Treatment with zanubrutinib was initiated after the diagnosis of an IgM-secreting CD5-/CD10- B-cell non-Hodgkin lymphoma, as indicated OCT: optical coherence tomography; RNFL: retinal nerve fiber layer

At his initial neuro-ophthalmology evaluation, approximately one month after symptom onset, the patient denied pain with eye movements or visual symptoms in the left eye. He reported no known family history of vision loss. Best-corrected visual acuity measured 20/50 in the right eye and 20/20 in the left eye. Examination demonstrated a right relative afferent pupillary defect. Funduscopic examination revealed grade 2 right optic disc edema, with circumferential obscuration of the disc margins, and a cup-to-disc ratio of 0.35. The left optic nerve appeared healthy, without edema or pallor, and had a cup-to-disc ratio of 0.45. Humphrey visual field testing demonstrated severe depression in the right eye (mean deviation, −23.23 dB) and a minimal superior arcuate defect in the left eye (mean deviation, −3.50 dB). OCT demonstrated RNFL thicknesses of 115 µm and 106 µm in the right and left eyes, respectively.

Although funduscopic examination continued to demonstrate right optic disc edema, the right RNFL thickness had substantially decreased from 223 µm at the preceding general ophthalmology evaluation, suggesting interval resolution of the edema (Figure 1). Given the patient's age and presentation, giant cell arteritis (GCA) was considered. He denied headache, jaw claudication, scalp tenderness, polymyalgia, or weight loss. The erythrocyte sedimentation rate (ESR) was mildly elevated at 33 mm/hr (reference range, 0-20 mm/hr), whereas C-reactive protein (CRP) was 2.51 mg/L (reference range, < 3 mg/L), and the platelet count was 239 × 10³/mm³ (reference range, 119-332 × 10³/mm³). Temporal artery biopsy or ultrasonography was not performed. Additional laboratory evaluation for infectious and autoimmune etiologies was unrevealing (Table 2).

**Table 2:** Infectious and autoimmune laboratory evaluation during the initial diagnostic workup ANA: antinuclear antibody; ANCA: antineutrophil cytoplasmic antibody; MPO: myeloperoxidase; PR3: proteinase 3; HSV: herpes simplex virus; VZV: varicella-zoster virus; IgG: immunoglobulin G; PCR: polymerase chain reaction

Serological test	Result	Interpretation
ANA by IFA	Negative	Negative
ANCA profile (MPO, PR3, C-ANCA, P-ANCA, atypical P-ANCA)	Negative	Negative
Lyme (Borrelia burgdorferi) PCR	Negative	Negative
Bartonella DNA PCR	Negative	Negative
HSV-1 IgG	Nonreactive	Negative
HSV-2 IgG	Nonreactive	Negative
VZV IgM	<0.91	Negative
Syphilis serology	Negative	Negative

The patient was subsequently lost to follow-up. About one year later, he returned with new vision loss in the previously unaffected left eye. On examination, there was diffuse optic disc pallor in the right eye and new optic disc edema in the left eye. Best-corrected visual acuity was 20/40 in the right eye and 20/50 in the left eye, with severe bilateral visual field depression (mean deviations of -25.81 dB and -28.44 dB, respectively). OCT showed marked RNFL thinning in the right eye (35 µm) and increased RNFL thickness in the left eye (126 µm), consistent with new optic disc edema. Over the following months, the patient experienced progressive bilateral vision loss with sequential optic atrophy despite repeatedly unremarkable orbital MRI studies. Serial neuro-ophthalmic examinations showed progressive bilateral optic disc and neuroretinal rim pallor with severe visual field constriction and RNFL thinning.

CT angiography (CTA) of the head and neck revealed approximately 70% stenosis of the left internal carotid artery. MRI of the brain and orbits with and without contrast showed normal bilateral orbits without evidence of an optic nerve lesion or other compressive abnormality (Figure 2). The patient denied constitutional symptoms, including fever, night sweats, and unintentional weight loss. As part of the expanded systemic evaluation following fellow-eye involvement, serum protein electrophoresis was obtained for the first time during this patient's workup and revealed a monoclonal M-spike of 0.6 g/dL (normal, 0 g/dL). Cerebrospinal fluid analysis was considered but not performed because the patient deferred lumbar puncture.

**Figure 2:**
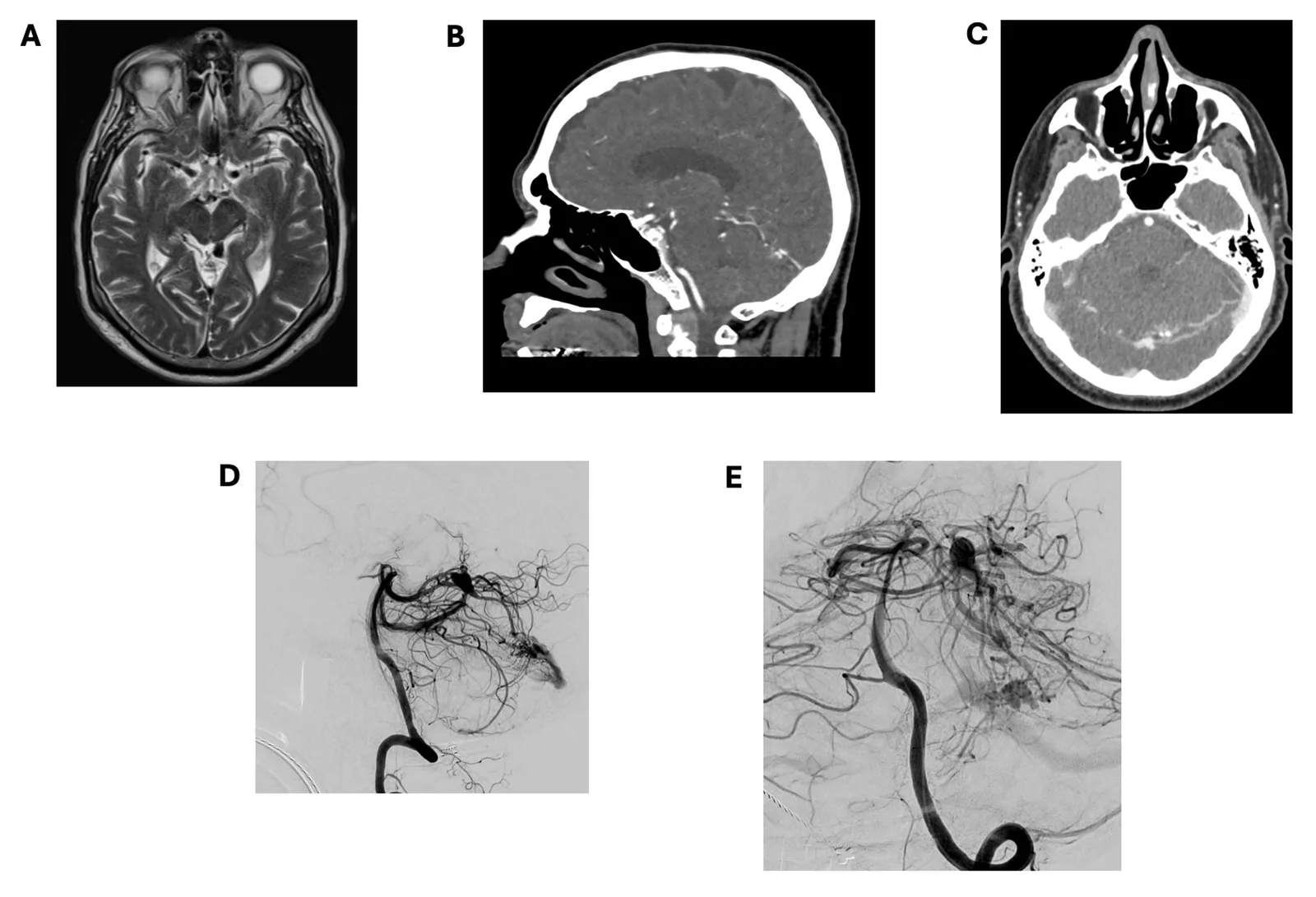
Neuroimaging obtained during the evaluation of progressive optic neuropathy (A) Axial T2-weighted MRI of the brain. MRI of the brain and orbits did not demonstrate an optic nerve lesion or other compressive abnormality to account for the patient's visual loss. (B-C) CTA of the head demonstrating the vascular anatomy in complementary sagittal and axial views. (D-E) Catheter cerebral angiography demonstrating the left transverse sinus dural AV fistula supplied by branches of the left superior cerebellar artery, with associated feeding-artery aneurysmal abnormality and venous shunting. The patient subsequently underwent embolization of the fistula and coil embolization of the aneurysm; however, his visual symptoms did not improve, prompting continued investigation for an alternative etiology MRI: magnetic resonance imaging; CTA: computed tomography angiography; AV: arteriovenous

Given the presence of a monoclonal gammopathy, the patient underwent hematologic evaluation. Bone marrow biopsy demonstrated involvement by CD5-/CD10- B-cell non-Hodgkin lymphoma involving approximately 20-25% of marrow cellularity, with several interstitial and paratrabecular lymphoid aggregates. There was no morphologic evidence of Waldenström macroglobulinemia, and cytogenetic studies, fluorescence in situ hybridization, and MYD88 mutation testing were negative. Additional laboratory evaluation demonstrated an elevated IgM level (1,023 mg/dL), elevated serum free kappa light chains, and an M-spike of 0.9 g/dL (Table 3).

**Table 3:** Relevant laboratory findings during hematologic evaluation IgM: immunoglobulin M; LDH: lactate dehydrogenase

Laboratory test	Result	Reference range
IgM	1,023 mg/dL	15–143 mg/dL
Serum free kappa light chains	80.1 mg/L	3.3–19.4 mg/L
Serum free lambda light chains	9.4 mg/L	5.7–26.3 mg/L
Kappa/lambda free light-chain ratio	8.52	0.26–1.65
M-spike	0.9 g/dL	Not observed
Serum viscosity	1.7 relative to saline	1.4–2.1
LDH	153 IU/L	121–224 IU/L
Beta-2 microglobulin	1.9 mg/L	0.6–2.4 mg/L

The patient was initiated on zanubrutinib therapy. Serial laboratory studies demonstrated declining IgM levels (Figure 3) and resolution of the clonal B-cell population on flow cytometry. At post-treatment follow-up, the patient reported subjective improvement in daytime vision. Best-corrected visual acuity was 20/40−1 in the right eye and 20/30 in the left eye; however, visual fields remained severely depressed, with mean deviations of −29.51 dB and −30.14 dB, respectively. Neuro-ophthalmic examination continued to demonstrate severe bilateral optic atrophy. 

**Figure 3:**
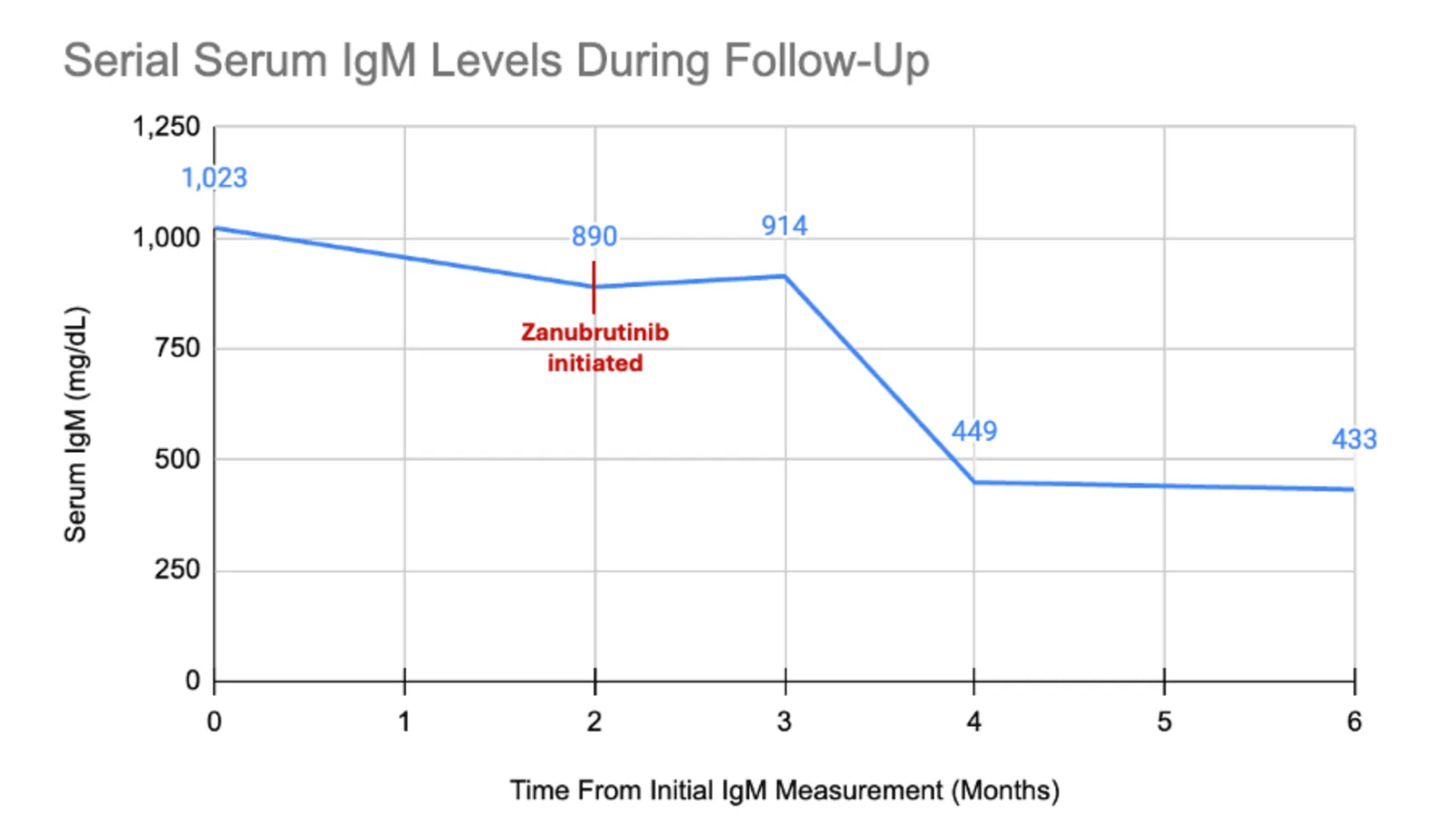
Serial serum IgM levels during follow-up Serum IgM levels are shown relative to the initial measurement (Month 0). Zanubrutinib was initiated at approximately Month 2, as indicated. The laboratory reference range was 15-143 mg/dL IgM: immunoglobulin M

## Discussion

We describe the case of a patient with sequential bilateral optic neuropathy developing over approximately one year, which ultimately led to the diagnosis of an IgM-secreting CD5-/CD10- B-cell non-Hodgkin lymphoma. The diagnosis was particularly challenging because of repeatedly unrevealing orbital imaging, the absence of constitutional symptoms, and incidental vascular abnormalities that initially appeared to explain the patient's visual decline. Although NAION remained an important consideration, the absence of a classic “disc-at-risk” appearance in the fellow eye and the subsequent clinical course prompted consideration of alternative etiologies.

Lymphoma-associated optic neuropathy is an uncommon but well-recognized neuro-ophthalmic manifestation of hematologic malignancy. Optic nerve involvement may occur in isolation, in association with systemic lymphoma or central nervous system disease, or as part of primary intraocular lymphoma, reflecting the diverse pathways through which lymphoma may affect the visual system [2]. Neurologic manifestations may result from direct lymphomatous infiltration, leptomeningeal dissemination, vascular compromise, paraneoplastic phenomena, or treatment-related complications [5]. Consequently, the clinical presentation is highly variable, and no single clinical, radiographic, or laboratory finding reliably establishes the diagnosis. Instead, evaluation often requires the integration of ophthalmic examination, neuroimaging, cerebrospinal fluid analysis, systemic evaluation, and, in selected cases, tissue biopsy [2,6-7]. Because delayed recognition may result in irreversible visual loss, suspected leukemic or lymphomatous optic nerve involvement should be approached as a neuro-oncologic emergency [7].

The precise mechanism of optic neuropathy in our patient remains uncertain. Direct lymphomatous infiltration appears less likely given two unrevealing orbital MRI examinations and the absence of radiographic evidence of central nervous system involvement. However, normal orbital imaging does not exclude lymphoma-associated optic neuropathy. Several reports have described clinically significant optic nerve involvement despite initially normal or minimally abnormal MRI findings, suggesting that early infiltrative disease or indirect mechanisms may evade conventional imaging [8-9]. Likewise, leptomeningeal disease was considered less likely because the patient lacked additional neurologic manifestations and neuroimaging remained unrevealing.

Cerebrospinal fluid analysis was not performed because the patient deferred lumbar puncture. Although the patient's vascular risk factors and carotid artery disease may have contributed to ischemic optic nerve injury, NAION remained an important competing diagnosis given his age, vascular risk factors, painless visual loss, and initial optic disc edema with hemorrhage. The subsequent clinical course prompted evaluation for alternative etiologies; however, NAION could not be definitively excluded. Furthermore, the identification of a monoclonal gammopathy and indolent B-cell lymphoproliferative disorder during an extensive systemic evaluation does not establish a causal relationship with the optic neuropathy, and coincidental co-occurrence remains possible.

An alternative explanation is that the optic neuropathy was related to the patient's underlying IgM-secreting lymphoproliferative disorder through an indirect mechanism. Previous reports of IgM-associated disorders, particularly Waldenström macroglobulinemia, have described optic neuropathy in the absence of radiographic evidence of optic nerve infiltration, with proposed mechanisms including paraproteinemic, immune-mediated, and hyperviscosity-related injury rather than direct neoplastic invasion [8-9]. Although our patient's bone marrow findings and negative MYD88 mutation testing were not consistent with Waldenström macroglobulinemia, an indirect relationship between the lymphoproliferative disorder and optic neuropathy remained possible. The patient reported subjective improvement in daytime vision following the initiation of zanubrutinib; however, objective visual fields remained severely depressed.

Both optic nerves had already developed severe RNFL thinning by the time treatment was initiated, and a floor effect associated with advanced RNFL loss limits interpretation of subsequent structural stability. Taken together, the relationship between the lymphoproliferative disorder and optic neuropathy remains associative rather than definitively causal. The identification of an IgM-secreting B-cell lymphoproliferative disorder and previously described mechanisms of IgM-associated optic nerve injury support a possible relationship. However, the NAION-compatible initial presentation, normal serum viscosity, absence of radiographic evidence of direct optic nerve infiltration, lack of cerebrospinal fluid evaluation, and RNFL floor effect limit causal inference. Coincidental co-occurrence therefore cannot be excluded, and the available clinical data do not permit the determination of a definitive pathogenic mechanism. Hereditary optic neuropathies were considered less likely given the patient’s advanced age at presentation and the absence of a known family history of vision loss, but they cannot be definitively excluded without genetic testing.

This case also illustrates the diagnostic challenges encountered when atypical optic neuropathy occurs in the setting of multiple competing diagnoses. Identification of a dural AV fistula and left carotid artery stenosis represented plausible explanations for the patient's visual symptoms and appropriately redirected the diagnostic evaluation. However, continued visual deterioration, sequential involvement of the fellow eye, and repeatedly unrevealing orbital imaging prompted reconsideration of the differential diagnosis. Published reports similarly emphasize that lymphoma-associated optic neuropathy may mimic more common inflammatory, ischemic, or compressive optic neuropathies, particularly early in the disease course, contributing to delays in diagnosis and potentially irreversible visual loss [2,6,8,10].

Several important clinical lessons emerge from this case. First, progressive sequential bilateral optic neuropathy should prompt continued diagnostic evaluation when the clinical course is not adequately explained by more common etiologies. Second, repeatedly unrevealing orbital MRI findings should not preclude consideration of lymphoma-associated optic neuropathy or other systemic hematologic disorders when clinical suspicion persists. Because leukemic or lymphomatous optic nerve involvement may require urgent treatment, continued investigation is warranted when the clinical course remains unexplained despite nondiagnostic initial testing [7].

Finally, hematologic evaluation, including assessment for monoclonal gammopathy and an underlying lymphoproliferative disorder, should be considered in selected patients with progressive, otherwise unexplained optic neuropathy. Early multidisciplinary collaboration among neuro-ophthalmology, hematology, and neuroradiology may facilitate earlier diagnosis and initiation of disease-directed therapy before irreversible visual loss occurs.

## Conclusions

This report highlights sequential bilateral optic neuropathy occurring in association with an IgM-secreting CD5-/CD10- B-cell non-Hodgkin lymphoma detected during an expanded systemic evaluation. The diagnostic evaluation was complicated by two unrevealing orbital MRI examinations and competing vascular abnormalities that initially provided potential alternative explanations for the patient’s visual decline. Although a relationship between the lymphoproliferative disorder and optic neuropathy is biologically plausible, the available clinical data do not establish causation or a definitive mechanism of optic nerve injury, and competing etiologies, including NAION, could not be completely excluded. Clinicians should maintain a broad differential diagnosis when evaluating progressive, sequential optic neuropathy that remains unexplained following the initial workup. Nondiagnostic orbital imaging should not preclude continued systemic investigation when the clinical course remains unexplained. In selected patients, hematologic evaluation, including serum protein electrophoresis, may identify an underlying monoclonal gammopathy or lymphoproliferative disorder. Multidisciplinary collaboration among neuro-ophthalmology, hematology, and neuroradiology may facilitate a comprehensive diagnostic approach to these diagnostically challenging cases.
